# Protective effect of zinc-hydroxyapatite toothpastes on enamel erosion: An *in vitro* study

**DOI:** 10.4317/jced.53068

**Published:** 2017-01-01

**Authors:** Claudio Poggio, Chiara Gulino, Maria Mirando, Marco Colombo, Giampiero Pietrocola

**Affiliations:** 1 Department of Clinical-Surgical, Diagnostic and Pediatric Sciences, Section of Dentistry, University of Pavia, Pavia, Italy

## Abstract

**Background:**

The aim of the present study was to test the impact of different toothpastes with Zinc-Hydroxyapatite (Zn-HAP) on preventing and repairing enamel erosion compared to toothpastes with and without fluoride.

**Material and Methods:**

The following four toothpastes were tested: two toothpastes with Zn-HAP, one toothpaste with fluoride and one toothpaste without fluoride. An additional control group was used in which enamel specimens were not treated with toothpaste. Repeated erosive challenges were provided by immersing bovine enamel specimens (10 per group) in a soft drink for 2 min (6mL, room temperature) at 0, 8, 24 and 32 h. After each erosive challenge, the toothpastes were applied neat onto the surface of specimens for 3 min without brushing and removed with distilled water. Between treatments the specimens were kept in artificial saliva. Enamel hardness, after the erosive challenge and toothpaste treatment was monitored using surface micro-hardness measurements.

**Results:**

As expected, repeated erosive challenge by a soft drink for total of 8 min significantly reduced enamel surface hardness (ANOVA, *p* < 0.05). No re-hardening of the surface softened enamel was observed in the group treated with fluoride-free toothpaste. Surface hardness of the softened enamel increased when the specimens were treated with the fluoride toothpaste and the two toothpastes with Zn-HAP (*p* < 0.05).

**Conclusions:**

Toothpaste with Zn-HAP resulted in significant enamel remineralisation of erosively challenged enamel, indicating that these toothpastes could provide enamel health benefits relevant to enamel erosion.

** Key words:**Enamel, erosion, remineralization, surface hardness, toothpastes.

## Introduction

Dental erosion has been defined as the chemical dissolution of the hard tissues by acids having non-microbiological origin (1). Dental erosion is a common problem in modern societies due to the increased consumption of acidic drinks, such as soft drinks, sport drinks, fruit juices, which have a high potential to cause enamel demineralization (2). In previous times dental erosion in the cervical area were reported mostly in elderly people. However, dietary changes and inadequate oral hygiene have led to enamel erosion becoming more frequent among young people. The development of erosion involves a chemical process in which the inorganic phase of the tooth is demineralized, thereby reducing the hardness of the tooth substance. Subsequent mechano-abrasive challenges, for example through tooth brushing, results in the loss of tooth substance (3).

Dental enamel consists of 95% calcium hydroxyapatite, 4% water and about 1% organic material. Enamel is organized by and large in prisms; however, aprismatic enamel with a thickness of up to 100 microns has been reported at the enamel surface, which is generally more highly mineralized than subsurface enamel. The early stages of dental erosion are characterized by a softening of the enamel surface to a depth of the order of 0.5 to 10 microns. Many studies have been carried out to understand if the process of enamel demineralization at the early stage is reversible (4). Biological and chemical factors in the oral environment influence the progress of enamel softening and erosion. Saliva provides protective effects by neutralizing and clearing dietary acids; it is also a source of inorganic ions necessary for the remineralization process (5). Enamel has no spontaneous biological capability to be repaired when affected by specific dental pathologies such as caries, abrasions or fractures because it contains no cells (6). The loss of substance by erosion is a cyclic and dynamic process with periods of demineralization and remineralization. Thus, preventive measures against erosion are required.

Toothpastes have been considered effective and accessible vehicles to improve enamel resistance to erosive attacks (7). Many types of toothpaste recently introduced are claimed to prevent erosion. Fluoride dentifrices have been shown to have some protective effect against the erosive challenge of a cola drink *in vitro* (8). However, conventional fluoride-containing toothpastes do not appear to be able to protect sufficiently well against erosive challenges (9). New toothpastes formulations have therefore been developed to provide more effective protection from dietary acids and hence effective protection against enamel erosion.

BioRepair Plus (Coswell S.p.A., Bologna, Italy) is fluoride free toothpaste, which includes zinc-hydroxyapatite micro-particles (Zn-HAP). The zinc-hydroxyapatite micro-particles (Microrepair®) are biomimetic to the mineral that forms enamel. Their mode of action in the context of erosion is based on their ability to be delivered to and adhere to natural enamel tissue (10). It is postulated that they are able to form a sacrificial layer of calcium phosphate mineral thus protecting the underlying enamel from damage by dietary acids and provide a localized, targeted source of calcium and phosphate to repair demineralized enamel.

Softening of the enamel surface is an early manifestation of the erosion process. Reduced surface hardness, which accompanies erosion of the enamel surface by acidic beverages, can be assessed using a physical measurement such as the hardness tests (11). Hardness is defined as the resistance of a material to indentation or penetration. It has been used to predict the wear resistance of a material and its ability to abrade or be abraded by opposing tooth structures.

The aim of the present study was to test the impact of different toothpastes with Zinc-Hydroxyapatite (Zn-HAP) on preventing and repairing enamel erosion compared to toothpastes with and without fluoride.

## Material and Methods

The *in vitro* study reported here utilized a cyclic demineralization/remineralization model in which bovine enamel specimens were exposed to an erosive challenge, toothpaste treatment and storage in artificial saliva at the start (0h) and after 8h, 24h and 32h.

The following four toothpastes were tested.

- toothpaste without fluoride (Subito/Incos Cosmeceutica s.r.l., 40050 Funo, Italy),

- fluoride toothpaste, 1450 ppm F- as NaF (Eufresh/CIO Farmaceutici s.r.l., 81100 Caserta, Italy),

- toothpaste with Zn-HAP (Microrepair®), without fluoride (Biorepair/Coswell S.P.A., 40050 Funo, Italy),

- toothpaste with Zn-HAP (Microrepair®) and zinc pyrrolidone carboxylic acetate (Zn-PCA), without fluoride (Biorepair Plus/Coswell S.P.A., 40050 Funo, Italy).

▪Specimen preparation 

Enamel specimens were prepared from fifty freshly extracted bovine permanent mandibular incisors. Teeth had to be free of cracks, hypoplasia and white spot lesions. After extraction, teeth were cleaned to remove soft tissue and stored in a solution of 0.1% (wt/vol) thymol. The enamel specimens were cut at the enamel-dentin junction with a high-speed diamond rotary bur with a water-air spray. The samples were placed into Teflon molds measuring 10 x 8 x 2 mm and embedded in self-curing, fast-setting acrylic resin (Rapid Repair, DeguDent GmbH, Hanau, Germany) in such a way that the exposed buccal surface was plano-parallel to the bottom of the mold.

▪Experimental Groups

There were five experimental groups: A: erosive challenge, no toothpaste treatment, B: erosive challenge + non-fluoride toothpaste treatment, C: erosive challenge + fluoride toothpaste treatment, D: erosive challenge + Zn-HAP toothpaste treatment, E: erosive challenge + Zn-HAP, Zn-PCA toothpaste treatment.

▪Erosive Challenge

A popular soft drink (Coca Cola / Coca Cola Company, Milano, Italy) was chosen for the erosive challenge. The pH at 20˚C, buffering capacity, concentration of calcium and phosphate of the beverage were measured (12). Measurements were performed in triplicate and average values calculated.

The specimens were immersed in 6mL of the soft drink for 2 min at room temperature before rinsing with deionized water. Four erosive challenges were carried out at 0, 8, 24 and 32 h, hence for a total of 8 minutes (11).

▪Toothpaste Treatment 

The toothpastes were applied neat onto the surface of the specimens without brushing to cover the entire surface and removed after 3 minutes by washing with distilled water. The toothpastes were applied at 0, 8, 24 and 32 h (11) immediately after the erosive challenge.

▪Specimen Storage

The enamel specimens were stored in artificial saliva (pH 7.0, 14.4 mM NaCl; 16.1 mM KCl; 0.3m mM Cl2.6H20; 2.9 mM K2HPO4; 1.0 mM CaCl2.2H2O; 0.10 g/100 ml sodium carboxymethylcellulose) (13) between the erosive challenge/ toothpaste treatment sessions.

▪Surface micro-hardness (SMH) measurements

The micro-hardness of the enamel surface was measured at the start (0h) and at the end of the experimental procedure (32h). The micro-hardness tester (Galileo Isoscan HV1 OD; LTF SpA, Antegnate, BG, Italy) was equipped with a Vickers diamond indenter. The indenter was applied with a load of 50g for 10 seconds and a 40x objective lens was used to measure the diagonal indentation length. Five indentations were made on the surface of each specimen; they were equally placed in a circle, each no closer than 0.5 mm to adjacent indentations. Indentation length was converted to Vickers Hardness (HV) using the following equation: HV = 1.854 P /d 2, where HV is Vickers Hardness in kgf/mm2, P is the load in kgf and d is the length of the diagonals in mm. For a given specimen, the five hardness values for each surface were averaged and reported as a single value.

▪Statistical analysis

Population size, margin of error, confidence level and standard deviation were set to determine the sample size. The data were analyzed using Stata 12 software (Stata, College Station, Texas, USA). Descriptive statistics including the mean, standard error of mean, and minimum and maximum values were calculated for all groups. Statistical analysis of the results of micro-hardness testing included Shapiro-Wilk test to assess the normality of the distributions followed by non-parametric Kruskal-Wallis analysis of variance (ANOVA) and Mann-Whitney U comparison test. A significant level of α = 0.05 was set for comparison between the groups.

## Results

**Figure 1:**
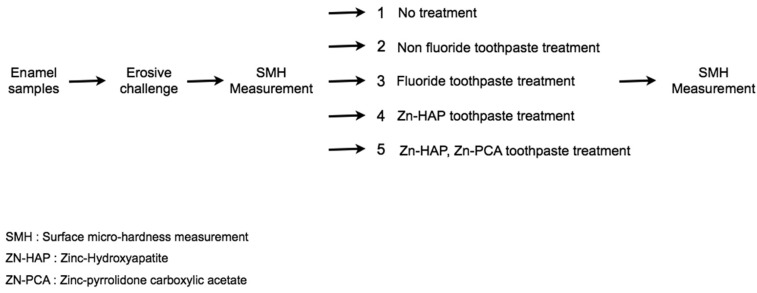
Flow-chart of the experimental phases of the study.

**Figure 2:**
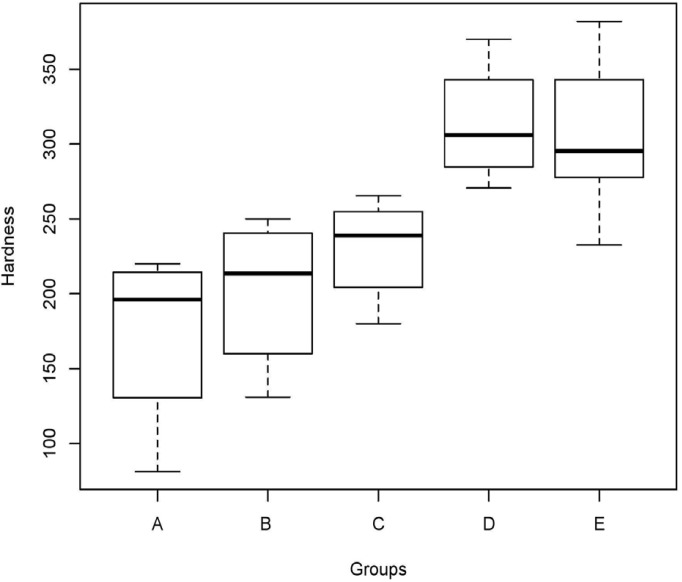
Mean ± SD of the enamel hardness (kgf/mm2) of the five tested groups and of intact enamel.

Descriptive statistics of the enamel surface hardness in terms of VH of each group are presented in  Table 1 and figure 1. The mean enamel surface hardness of sound enamel (0h) was 291.4 (SD=18.4) with a median of 213.5; there were no significant differences between the experimental groups (ANOVA, *p* = 0.30). The erosive challenge from four 2-minute exposures of the enamel specimens to a soft drink (Group A) significantly reduced enamel surface hardness (ANOVA, *p*<0.05). Treating the enamel specimens with non-fluoride toothpaste (Group B) did also resulted in a significant reduction of enamel hardness (*p* < 0.05). Surface hardness of the erosively challenged enamel increased significantly (*p* < 0.05) when treated with a standard fluoride toothpaste (Group C) or with non-fluoride toothpaste containing Zn-HAP (Groups D and E) compared to both Groups A (no toothpaste treatment) and B (treatment with non-fluoride toothpaste). No significant differences were found between sound enamel and remineralized enamel (*P* > 0.05) after treatment with fluoride toothpaste (Group C) or with non-fluoride toothpaste containing Zn-HAP (Groups D and E), (Fig. 2).

**Table 1:**
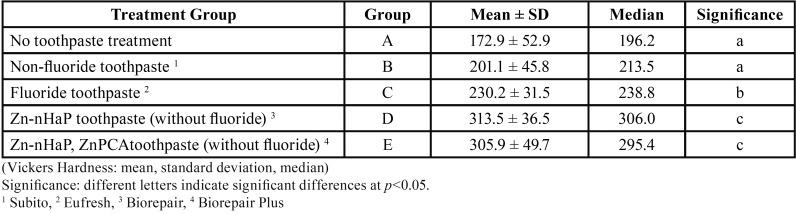
Enamel hardness after four cycles of erosive challenge and toothpaste treatment.

## Discussion

This study has confirmed that Zn-HAP toothpaste without fluoride is able to counteract the erosive effect of an acidic soft drink on dental enamel and in fact lead to remineralization of surface softened enamel as shown by a statistically significant increase in enamel hardness.

The hardness values of sound enamel measured in this study were in line with those reported in the literature. Zanet *et al.* (13), reported values of approximately 230 VH, and Al-Jobair (14) measured values of about 350 VK; in both studies the measuring conditions were similar to those used in the present study.

The experimental design applied in this study utilized a demineralization/remineralization cycling model. Models of this type have been shown to be more relevant for the study of preventive measures against demineralized enamel (such as erosive damage or early caries) compared to single application studies because they resemble more closely the reality of enamel exposure in the mouth.

In the oral environment, host factors (such as the mineral concentration of the tooth, and the pellicle and plaque formation) can influence the progression of demineralization (15-17).

Salivary factors, such as the salivary flow rate, composition and buffering capacity, might exert protective action on dental surface (6,15,18). For this reason, a further step was taken in this study to enhance the relevance of the model by storing specimens in artificial saliva between the experimental procedures.

This step may have contributed to the increase in enamel hardness found in the specimens treated with non-fluoride toothpaste (Group B), and also provided some degree of remineralization in the other treatment groups (Groups C to E). Despite this, it is apparent from the results presented that fluoride toothpaste (Group C) and Zn-HAP toothpaste without fluoride were able to increase significantly enamel hardness despite the repeated erosive challenge form 2 minute exposure of the specimens to an acidic soft drink.

As expected, the erosive challenge from immersing the specimens four times for 2 minutes (8 min total) in an acidic soft drink significantly reduced enamel surface hardness (ANOVA, *p* < 0.05). The drop in surface hardness in similar to that reported in the literature and confirms the suitability and relevance of this approach.

The influence of the toothpastes on the prevention of demineralization observed in the present study would be clinically beneficial. In this study no significant differences between the remineralizing effects of the Zn-HAP toothpastes (without fluoride), and the fluoride toothapste with 1450 ppm F - were found. Both technical approaches were able to maintain/restore enamel hardness following repeated erosive challenges. In the case of the Zn-HAP technology, this indicates that supplying calcium-phoshate minerals which are reatined on the enamel surface is a suitable and effective route to counteract the effect of an erosive challenge. The data suggest that the mode of action is a combination of reducing the demineralization effect of the acidic challenge and a remineralisation/repair effect brought about by the extra provison of calcium and phosphates.

In this study two toothpastes with Zn-HAP were evaluated. The toothpaste marketed as Biorepair Plus also contained the zinc salt zinc pyrrolidone carboxylic acetate, which delivers free zinc ions to provide antibacterial efficacy. The reason this product was included in this study was because zinc ions have been described as a crystallization inhibitor and have therefore been used in toothpaste as anti-calculus agents in addition to their use as an anti-bacterial active (19). It was therefore important to assess whether a Zn-HAP toothpaste with Zn-PCA would reduce the protective efficacy of a Zn-HAP toothpaste without Zn-PCA. The results presented in  Table 1 clearly show that both toothpastes showed very similar effects and it can therefore be concluded that the addition of Zn-PCA to Zn-HAP toothpaste does not reduce the protective effect against an erosive challenge.

In conclusion, toothpaste with Zn-HAP (Biorepair, Biorepair plus) resulted in significant enamel remineralisation of erosively challenged enamel, indicating that these toothpastes could provide enamel health benefits relevant to enamel erosion.
